# The Southern Dental Association—Abstract of 1896

**Published:** 1897-03

**Authors:** J. M. Walker


### The Southern Dental Association—Abstract of 1896.

Reported for the DENTAL REGISTER by Mrs. J. M. WALKER.

The Southern Dental Association held its twenty-seventh annual session in Asheville, N. C., July 28th, 29th and 30th, 1896.

Owing to the unfortunate circumstances which necessitated changing the date of meeting from November to July, giving the committees insufficient time in which to prepare for the meeting, and the double change of place (from Nashville to Lookout Inn and from Lookout Inn to Asheville), the meeting was not the usual success in point either of attendance or number of papers presented.

The meeting was held in the ball-room of the Battery Park Hotel.

The Association was called to order Tuesday, July 28th, at 11 A. M., with the President, Dr. John S. Thompson, of Atlanta, Ga., in the chair.

After prayer by the Rev. Dr. H. F. Chreitzberg, an address of welcome was delivered by Mr. Lawrence P. McLoud, of Asheville, and responded to by Dr. J. Y. Crawford, of Nashville, Tenn., on behalf of the Association.

The President then read his Annual Address, an interesting *resume* of the history of the Association from its organization to the present meeting.

A vote of thanks was tendered Dr. Thompson for his labor in searching the records and compiling this interesting history, which was ordered published in full in the Transactions.

### CASTING FILLINGS AND ABUTMENTS FOR BRIDGES.

DR. C. L. ALEXANDER, of Charlotte, N. C., read a paper describing his method of dealing with a class of teeth that can not be satisfactorily treated by the old method of filling and crowning, a method by means of which results can be accomplished in an artistic and beautiful manner in one-half the time required by the old method. The fundamental principle is an old one—that of fusing and flowing metal over platinum-sheet or foil which has been burnished over the surface, to be restored, with holes for retaining post or posts. A little modeling compound is then heated over the spirit-lamp and pressed down firmly over the surface; when cool, the platinum-sheet and posts, in correct relation to each other, are removed, inverted, and the posts soldered to the platinum with pure gold. The piece is then placed in the mouth again, the foil trimmed and reburnished, and an impression taken, including an occlusion obtained by having the patient close the teeth together before the impression-material has become hard. The metal foundation will be drawn out by the impression-compound. The impression is then inverted and placed in an articulator, the contour of the tooth being built up with wax. Over the wax-surface gold or platinum foil is burnished, a suitable portion of the wax being left uncovered. Through this opening the wax is boiled out, leaving a matrix of metal in which is fused 20 or 22-karat-gold solder. The casting is then removed from the investment, finished up, and cemented to the tooth, and then given a final finish. For bicuspids and molars the cusps may be stamped up, using pure gold 35-gauge.

Abutments for bridges are made practically in the same way and placed in position upon the teeth, a model being thus secured from which the bridge is made in the usual way, the abutments being made of a higher-fusing metal than the bridge proper. By the use of these cast-abutments tooth-structure is saved in all cases, a display of gold from the labial aspect is avoided, and a light and substantial piece of work is secured, free from overhanging margins which afford a lodging-place for food-substance and a bed for the development of bacteria.

By this method the worst reclining teeth may be made to serve as abutment-piers without shaping up, provided only that all retaining-holes for bridge-posts be made parallel to each other.

The chief objection to this class of work is the possibility of the cement not standing, but the adaptation of the casting to the walls of the tooth, when properly made, is so perfect that little trouble is likely to be met with in this direction. Dr. Alexander added that he is now engaged in working upon cements, and hopes to succeed in making a cement that will be insoluble to such an extent that it will resist the action of the fluids of the oral cavity.

In the discussion of this paper Dr. A. P. Johnson, of Anderson, S. C., expressed the opinion that there is no cement now on the market that will hold this kind of work, especially that illustrated in bridge-abutments on inclined molars, and that he does not see the advantage of this over so shaping the teeth as to slip a band over the crown. In living teeth he would fear pulp-irritation from drilling the holes for the retaining-posts. For restoring incisors, however, he was much impressed with the method.

Dr. G. J. Friedricks considers this work, when it can be successfully accomplished, far preferable to an all-gold crown. The work, as represented by Dr. Alexander, is artistic, and its practical results represent the large contour fillings which are such a trial to both patient and operator.

Dr. S. W. Foster, of Atlanta, Ga., has done much work of a character quite similar to that of Dr. Alexander, though differing somewhat in details of construction, and has met with encouraging success in this method of restoring contour; has had to replace but one through failure of the cement. The secret of success lies in rolling the platinum, very thin, between copper plates, which takes away its elasticity and permits of obtaining very perfect margins. To drill the holes for the retaining-posts, and avoid pulp-irritation in living teeth, requires a thorough knowledge of tooth-anatomy.

Dr. J. Y. Crawford would use platinum for a matrix only for a porcelain inlay, using a gold matrix for a gold casting. He would also prefer the method of Dr. G. V. I. Brown, of Duluth, Minn., for large fillings, viz., filling the cavity one-third full of wax before taking the impression, replacing the wax with cement, into which the pins of the inlay are set, thus having a large body of cement next to the pulp and under the gold-filling casting or inlay. There is no deficiency of strength in this kind of work except that which arises from the susceptibility of the cement to the action of the oral fluids, which varies greatly in different mouths. It is not necessary to use very long pins. In bicuspids they can be located in the cusps.

Dr. C. Edmund Kells, Jr., of New Orleans, La., is not an advocate of bridge-work. Of every fifty cases presented in his practice, he declines forty-nine. When it *is* advisable, he prefers Dr. Parmly Brown's porcelain-work, anchored in fillings.

Dr. Crawford is especially opposed to anchoring bridges in in fillings, as they can rarely stand the strain of such leverage, and he challenges the moral force of Dr. Kell's views in regard to bridges. He cited several cases in which bridges had added greatly to the health, comfort and longevity of patients, enabling them to chew their food as they had not been able to do for years before.

Dr. R. K. Luckie, of Holly Springs, Miss., expressed himself as being very much impressed with Dr. Alexander's method, especially for badly broken-down bicuspids. If necessary, the tooth can be devitalized and the casting anchored by a post in the root, giving still greater strength. He also considers it very desirable for lateral incisors, which are difficult to crown because of the small, constricted neck. When nothing is left but the labial wall, a cast-filling of this kind, anchored in the root, would make a strong tooth and preserve the outer enamel face, which is so desirable. He thinks the cement should prove quite as durable in this work as in setting Logan crowns.

Dr. H. E. Beach is doubtful as to the desirability of this kind of work for bridge-abutments. The force of occlusion in bridge-work should be exerted in a direct line with the axis of the roots serving as abutments.

Dr. J. Y. Crawford wishes to emphasize the importance of exercising judgment in the careful study of each case that presents, to see which is best—of the various methods possible—carefully avoiding the danger of drifting into routine work. The great danger lies in specialism. Too many good dentists are inclined to avoid mechanical dentistry, throwing all work of that kind into the hands of the Cheap-John class. We need a better class of mechanical dentists—men who are artists as well as mechanics. He spoke at more length upon the very satisfactory results that can be obtained from swaging metal plates directly upon the plaster die. He said nothing can approximate a lower plate of aluminum swaged upon a plaster die, made with rubber attachments. It goes in like a wafer, and there will be no ulcerative absorption in the lower jaw. For setting all-gold crowns and bridge-abutments, Dr. Crawford recommends taking an impression and making a metal die of the tooth to be crowned, and fitting the crown upon that. When the crown is completed, let the patient wear it a day or two without cementing it on, to be sure that the occlusion, and the fit at the cervical margin, is all right. Then fill the crown with gutta-percha, warm it and slip it on and off the metal die, having oiled the latter slightly, removing all surplus of gutta-percha till you are sure you have just enough to set the crown properly ; then warm it again and place it in the mouth, and you will have saved your patient a great deal of suffering and annoyance.

DR. ALEXANDER prefers to smear the tooth with gutta-percha dissolved in chloroform, and then set the crown with cement.

Adjourned.

The morning session of the second day was devoted to clinics.

DR. C. EDWARD KELLS, JR., of New Orleans, and DR. GEO. H. WELLS, of Augusta, Ga., gave demonstrations of cataphoresis. In Dr. Kells' case the result was not entirely satisfactory, owing to the length of time consumed. The patient, a young lady, was very nervous, this being her first experience in the dental chair, and also unusually susceptible to the electric current, bearing only 3½ volts. After the expiration of fifty-five minutes the tooth, an upper central incisor, was sufficiently anæsthetized to permit of excavation for a cement filling.

DR. WELLS, in twenty minutes, using 10½ volts, anesthetized a very sensitive cavity in a lower bicuspid. He uses the Van Woert apparatus.

DR. C. L. ALEXANDER quite successfully injected eucaine for an extraction.

At the afternoon session a number of oral clinics were given. The patient for whom Dr. Hinman constructed the appliance described in the July (1896) issue of the *Dental Cosmos*, was present, wearing the appliance with great satisfaction, and was made the subject of an interesting lecture by DR. S. W. FOSTER.

DR. J. Y. CRAWFORD gave an oral clinic on a case presenting semi or half-prognathous features, with retained deciduous teeth, large accumulations of tartar, caries, hypertrophy of the gum, and general uncleanliness of the oral cavity. In regard to the baby-teeth, Dr. Crawford said : "I am often asked, 'What do you do with baby-teeth that have been too long retained?' I say, always take them out. This is the result of clinical observation. Always pull them out! It is the rule of Nature that there are twenty teeth to be shed and be replaced. If they remain in, contrary to God Almighty's will, pull them out. 'But,' you say, 'perhaps the permanent teeth will never erupt.' In forty-nine out of fifty cases they will erupt, and you don't want to sacrifice forty-nine for the sake of the other one. Remove them at approximately the proper age for them to be removed by nature—not according to the old English tables of eruption, however, for the time is being gradually retarded through the influence of our modern systems of education and diet. The teeth of American children do not erupt according to rule; the period is growing later and later. In the present case, after the removal of tartar, stumps, and baby-teeth, make him clean his mouth. And a word right here of clinical importance: Why do the teeth of the American human family decay as they do? Because they do not know how to clean the oral cavity. Give your patient a glass of water and tell him to rinse his mouth; he will sip it daintily and spit it out, as if he was afraid of it, and you will find that he has not washed out the blood, pumice-stone, and other debris. Give him more water, and a brush, and still he will not get it out. Teach your patients how to irrigate the oral cavity. Make them use elbow grease, common sense, and pure water. Show them how to irrigate the mouth: Throwing the head well back, passing the water through the inter-dental spaces and back to the fauces, gargling the throat. Why use pumice-stone, polishing powders, etc.? They only injure the gums. Water is Nature's great remedy. Give them a hand-mirror, and make them go to the window and examine the mouth thoroughly, so that they can see when it is really clean. Ninety-seven out of every hundred American citizens do not know how to clean their own mouths. When you get a new patient put him through this drill, so that he will know what it is to really clean the mouth. You prescribe an antiseptic, astringent mouth-wash, but after it has been used for a week or ten days the parts will become inured to it and cease to respond. Then you must change to something else; and, after a little while, change again. It is the same with topical applications as with systemic treatment—you expend the force of any given remedy after a time. For these sub-periosteal disturbances you will want aconite and iodine; you will recognize two very different types of cases. With mere local manifestations, use the officinal American preparation—the alcoholic tincture of iodine; but where the disease is more deeply located use Lugol's Solution. In all cases let this be your law: Eliminate all disease before you dismiss your patient. Do not temporize and leave some uncorrected abnormality to spread and breed continuance. I am astonished to see how many dentists do not clean the teeth of their patients, and do not teach them how to do it. If you prescribe hot water douches at night, you will be surprised at the improved condition next day."

Dr. C. E. Kells gave a clinical lecture on "The Use of Amalgam." He said: I use amalgam very satisfactorily, both to myself and to my patients. I see no use in spending hours in packing gold into molars where it is never seen—a most tedious operation to both operator and patient, and which fails comparatively soon. You may spend three, four, or five hours in making a beautiful operation. But to what end? Life is too short to waste so much time on such unnecessary work. It is far more satisfactory to expend but a fraction of the time, a fraction of your strength, to give but a fraction of discomfort to your patient, and yet accomplish the same result—saving the tooth—even more effectually, by filling the cavity with amalgam. Amalgam is my main dependence in a large class of cases. The less metal there is in a tooth, the better it is for the tooth. Fill the pulp-chamber with cement, and over that put a veneer of amalgam. You say amalgam will discolor the tooth? Yes, large masses of amalgam will do it, but don't use large masses. Use, partly, cement, as I have said, and in the anterior portion, where amalgam would show and not look well, combine gold and amalagm in the cavity. Fill the portion that will be visible with gutta-percha and complete the filling with amalgam. At the next sitting remove the gutta-percha, leaving a nice little cavity with one wall of amalgam, and fill that with gold. In bicuspids, when you do not want to put gold against the cervical wall, fill up to the gum-line with amalgam, and then complete the filling with gold. There is something peculiar in this combination that saves teeth better than all-gold or all-amalgam. You do your patients a kindness not to torture them with long sittings for gold-fillings in the back teeth. For the front teeth there is no choice, you have to use gold there. If you will take the same care in the use of amalgam that you do in using gold, you will get just as good results. I used to squeeze my amalgam through chamois, but I was troubled with the fibres. I now use China-silk—two thicknesses. I wash my amalgam in alcohol, and use Hardman's White Alloy.

Dr. J. Y. Crawford: What do you think about the liability to recurrence of decay in a mouth in which the front teeth are filled with gold and the back teeth with amalgam, as compared with a mouth in which only one metal is used, and that metal gold?

Dr. Kells: I think that if the presence of both gold and amalgam in the mouth has any special effect, that it is better for the teeth than all gold. That has been my clinical experience. Life is too short to put gold-fillings in the back teeth. If they were good teeth they would not require filling with anything. If they have large cavities they are not good teeth, and are not worth filling with gold.

At the close of lecture Dr. Kells exhibited specimens of teeth filled by his methods with amalgam, amalgam-and-cement, amalgam-and-gold, etc.

Dr. Alexander exhibited models of his cast-fillings, bridge-abutments, etc., illustrative of the paper read by him.

Dr. J. Y. Crawford described a case of what he called *dry socket*—a peculiar condition of the socket after tooth-extraction, which is accompanied with long-continued, persistent, severe pain, extending to the entire side of the face and head, throat and ear. In the case described the socket remained open and dry for a year, the extraction having been without hemorrhage, and with no pus or plasmic exudations whatever, but remaining persistently "clean and nice," tampons of gauze being removed after twenty-four hours, perfectly cleen and dry. Temporary relief was obtained by the insertion of cotton wet with chloroform and sweet-oil.

Dr. W. H. Richards described a similar case which he had seen in Atlanta, where it was being treated by Dr. Calhoun, the character of the case being evidently a novelty to him.

Dr. G. F. S. Wright has had two similar cases. Suspecting incipient necrosis, he treated with aromatic sulphuric acid and obtained relief in about two weeks.

Dr. H. E. Beach, of Clarksville, Tenn., has had a similar case, and concluded that the periosteal lining of the socket had been destroyed, leaving no surface from which exudation could take place. The patient described the pain from the sensitive, inflamed tissues, as "worse than four toothaches." By breaking down the alveolar socket in such cases we can induce a new circulation and fill up the socket with pabulum.

Dr. Crawford: In the case I mentioned, after twelve months I curetted the entire cavity, but at the end of another month there was no evidence of improvement. Should I have another case I think I shall break down the process entirely.

Dr. A. P. Johnson: In cases of difficult extraction, there is great strain upon the alveolar plate, and the pressure upon the soft tissues results in injury to the nerve-terminals. The distention of the parts is an abnormal condition, which interferes with normal repair work. To relieve the pain I press the sides of the socket hard together, having the plate sprung in. This pushing of the parts together gives great relief.

Dr. S. Ewing Smith, of St. Augustine, Fla.: No one has mentioned the method by which I obtained a cure in a very obtinate case, of the character described by Dr. Crawford, I had tried everything I had ever heard of, for weeks and months, without any sign of closing of the socket, which remained open, always of the same size, the patient suffering severe pain. Finally I procured a big nail—a 30-penny nail. I bent the point over, and throwing a cloth over the eyes of the patient, heated the nail red-hot and plunged the point into the socket! I made *a big burn*, and when I had cured the burn the original trouble had disappeared, and I heard no more of it.

DR. RICHARDS: In other words, "Give 'em fits, and then cure the fits!"

DR. G. J. FRIEDRICKS, of New Orleans, La., read a short paper on "Hygiene," of which the following is an abstract:

"The usual definition of hygiene, 'the art of preserving health,' is defective. It is more than an 'art,' it aims to increase and improve as well as to 'preserve.' The word 'health' is too vague to be of much value in this connection. In its broader sense, hygiene includes the examination of the conditions which affect the generation, development, growth and decay of individuals, of nations, and of races; being, on its scientific side, co-extensive with biology in its broadest sense, including sociology, rather than with physiology merely, as writers state. As regards practical hygiene—that is, the preventive of disease—we may seek to attain this end in two different ways, either by the endeavor to avoid or remove the causes of disease, or by making the body less susceptible to the action of these causes. But, considering the great number of causes of disease, and the impossibility of shunning them all, even with the greatest care, this very effort in itself becomes a cause of disease—witness the inscription on a tombstone in one of the cemeteries of Italy: 'In trying to be better, I am here!' On the other hand, in accordance with our recognized power in modifying plants and animals by regimen and breeding, it is probable that the human body might likewise be improved by a sort of hygiene organo-plasty—to use the phrase of Roger-Collard—and, at first sight, it appears strange that more attention is not paid to this branch of preventive medicine. To effect this it would be necessary to prevent the production of weak and unhealthy persons. Hygiene commences at the moment when two animated, wandering, microscopic molecules meet and mingle into that which is to grow into what you and I are, until its elements are given back to the cosmic storehouse whence they have been borrowed.

---

A VEGETABLE foreign body in the ear can be reduced in size before removal by instilling a few drops of glycerine or of alcohol and water.

